# Silicon via nutrient solution modulates deficient and sufficient manganese sugar and energy cane antioxidant systems

**DOI:** 10.1038/s41598-021-96427-z

**Published:** 2021-08-19

**Authors:** Kamilla Silva Oliveira, Renato de Mello Prado, Mirela Vantini Checchio, Priscila Lupino Gratão

**Affiliations:** 1 Laboratory of Plant Nutrition, Sector of Soils and Fertilizers, Department of Agricultural Production Sciences, São Paulo State University (UNESP), Via de Acesso Prof. Paulo Donato Castellane, s/n, Jaboticabal, São Paulo, 14884-900 Brazil; 2 Laboratory of Plant Physiology, Department of Applied Biology for Agriculture, São Paulo State University (UNESP), Via de Acesso Prof. Paulo Donato Castellane, s/n, Jaboticabal, São Paulo, 14884900 Brazil

**Keywords:** Plant stress responses, Plant sciences, Plant physiology

## Abstract

Manganese (Mn) is highly demanded by Poaceae, and its deficiency induces physiological and biochemical responses in plants. Silicon (Si), which is beneficial to plants under various stress conditions, may also play an important role in plants without stress. However, the physiological and nutritional mechanisms of Si to improve Mn nutrition in sugarcane and energy cane, in addition to mitigating deficiency stress, are still unclear. The objective of this study is to evaluate whether the mechanisms of action of Si are related to the nutrition of Mn by modulating the antioxidant defense system of sugarcane plants and energy cane plants cultivated in nutrient solution, favoring the physiological and growth factors of plants cultivated under Mn deficiency or sufficiency. Two experiments were carried out with pre-sprouted seedlings of *Saccharum officinarum* L. and *Saccharum spontaneum* L. grown in the nutrient solution. Treatments were arranged in a 2 × 2 factorial design. Plants were grown under Mn sufficiency (20.5 µmol L^−1^) and the deficiency (0.1 µmol L^−1^) associated with the absence and presence of Si (2.0 mmol L^−1^). Mn deficiency caused oxidative stress by increasing lipid peroxidation and decreasing GPOX activity, contents of phenols, pigments, and photosynthetic efficiency, and led to the growth of both studied species. Si improved the response of both species to Mn supply. The attenuation of the effects of Mn deficiency by Si depends on species, with a higher benefit for *Saccharum spontaneum*. Its performance is involved in reducing the degradation of cells by reactive oxygen species (21%), increasing the contents of phenols (18%), carotenoids (64%), proteins, modulating SOD activity, and improving photosynthetic and growth responses.

## Introduction

Manganese (Mn) is one of the most absorbed micronutrients by plants. Its biological importance is widely known. However, Mn deficiency in crops is evident in alkaline soils present in different regions of Europe and Asia^1^ or due to the excess of lime applied on the soil surface in tropical regions^2,3^, which increases the pH and decreases Mn availability in the soil.

Plants under Mn deficiency overproduce reactive oxygen species (ROS), such as superoxide radicals (O_2_^**·**-^), singlet oxygen (^1^O_2_), and hydrogen peroxide (H_2_O_2_). This leads to ROS accumulation and oxidative stress, which in turn cause lipid peroxidation^4,5^. Mn deficiency also reduces the contents of phenolic compounds^6^ and carotenoids^7^, which aid the defense system against ROS accumulation. In addition, the plant defense system can be stimulated by modulating the activity of enzymes such as superoxide dismutase (SOD)^5,8,9^ and peroxidases^3,5,10,11^. Mn deficiency causes damage to the thylakoid structure, decreasing the pigment content^12,13^ and the photosystem II quantum efficiency^14^, also contributing to ROS formation.

Furthermore, Mn deficiency may decrease the synthesis of proteins^15,16^ and consequently the production of dry matter of different plant species^5,17,18^.

The effects of Mn deficiency are little explored for sugarcane (*Saccharum officinarum* L.), although it is the second most extracted and exported micronutrient^19^ and is responsive to Mn application^20^.

Energy cane genotypes (*Saccharum spontaneum* L.) with high fiber contents have appeared recently. They are destined to energy production by burning^21^. This species has an advantage over sugarcane because it generates a higher ethanol production (from cellulosic fiber) and has longevity in field^21,22^. Mn is part of the metabolic pathway of the synthesis of phenols and lignin (fiber components)^6,23,24^ and could provide a higher benefit to this species. However, this effect is still unknown. The knowledge about the nutritional and physiological responses of *S. officinarum* L. and *S. spontaneum* L. to Mn supply or its deficiency is limited or inexistent, which is a matter of concern when aiming their optimum growth.

A strategy to enhance the response of sugarcane and energy cane to Mn application and to minimize the effects of its deficiency could be its joint application with silicon (Si). It may occur because Si is an element that benefits plant physiology and growth, especially under stressful conditions^25^ and in accumulating plants, such as sugarcane, which has a high root absorption of this element^26,27^.

Also, Si supply in the nutrient solution for sorghum promotes a reduction in lipid peroxidation by regulating the activity of the enzymes SOD and ascorbate peroxidase (APX); it also increases photosynthesis and consequently dry matter production in Mn-deficient plants, although there is no effect on Mn-sufficient plants^5^. Moreover, Si increases Mn use efficiency in sufficient and plants deficient in this micronutrient. Therefore, Si benefits physiological aspects by improving the conversion of the absorbed element into dry mass.

There is a possibility that Si may benefit Mn absorption because, according to^28^, the beneficial element activates H^+^-ATPases, and this carrier protein is involved with the transport primary of nutrients, including Mn^29^, and may increase its accumulation. Furthermore, there is influence of Si on the expression of IRT1 and AtNramp family transporter genes in rice^30^. These transporters act in the absorption and transport of Mn^31^ and have already been identified in sugarcane^32^. Studies have reported beneficial Si interactions in Mn accumulation in shoots and roots of several species, including wheat and corn, by increasing Mn transport^33^. Thus, Si could induce, at a transcriptional level, a super-expression of transporter protein genes, which could affect the accumulation of Mn in sugarcane and energy cane.

Another important benefit of Si is the increased production of non-enzymatic antioxidant compounds, such as phenols, as observed in wheat plants^34^. It increases the antioxidant response of plants and reduces the degradation of important organic compounds, such as chlorophyll, thus favoring photosynthesis. Chlorophyll degradation is a natural process^35^ that Mn deficiency may accelerate. Therefore, the Si effect delaying this process might benefit plants with or without manganese deficiency. However, the effects of Si may vary according to the Mn content in the plant and in cultivated species.

The hypotheses of this research are related to a need of knowing the mechanisms of action of Si related to Mn nutrition in sugarcane and energy cane. We hypothesize that the application of Si in the nutrient solution may increase Mn accumulation and decrease oxidative stress due to the increase in antioxidant compounds and consequently the increase in the chlorophyll content and the quantum efficiency of the photosystem II (PSII), thus improving production of dry matter of *S. officinarum* L. and *S. spontaneum* L. cultivated under sufficiency of Mn. In addition, these same beneficial effects of Si are evidenced in plants under Mn deficiency depending on the level of stress induced in species, which increases the efficiency of Mn use and decreases lipid peroxidation, thus favoring the physiological and growth aspects of crops.

If true, the hypotheses will reveal the physiological, biochemical, and nutritional relationship of Si with Mn. This enables proposing changes in the management of Mn application associated with Si aiming to improve nutrition with this micronutrient and favoring species growth with sustainability.

Thus, this study aims to evaluate whether Si can modulate the antioxidant defense system, favoring physiological, biochemical, nutritional, and growth aspects of two sugarcane species grown in a nutrient solution under Mn deficiency or not.

## Results

### Mn and Si accumulation and Mn use efficiency

**Figure 1:**
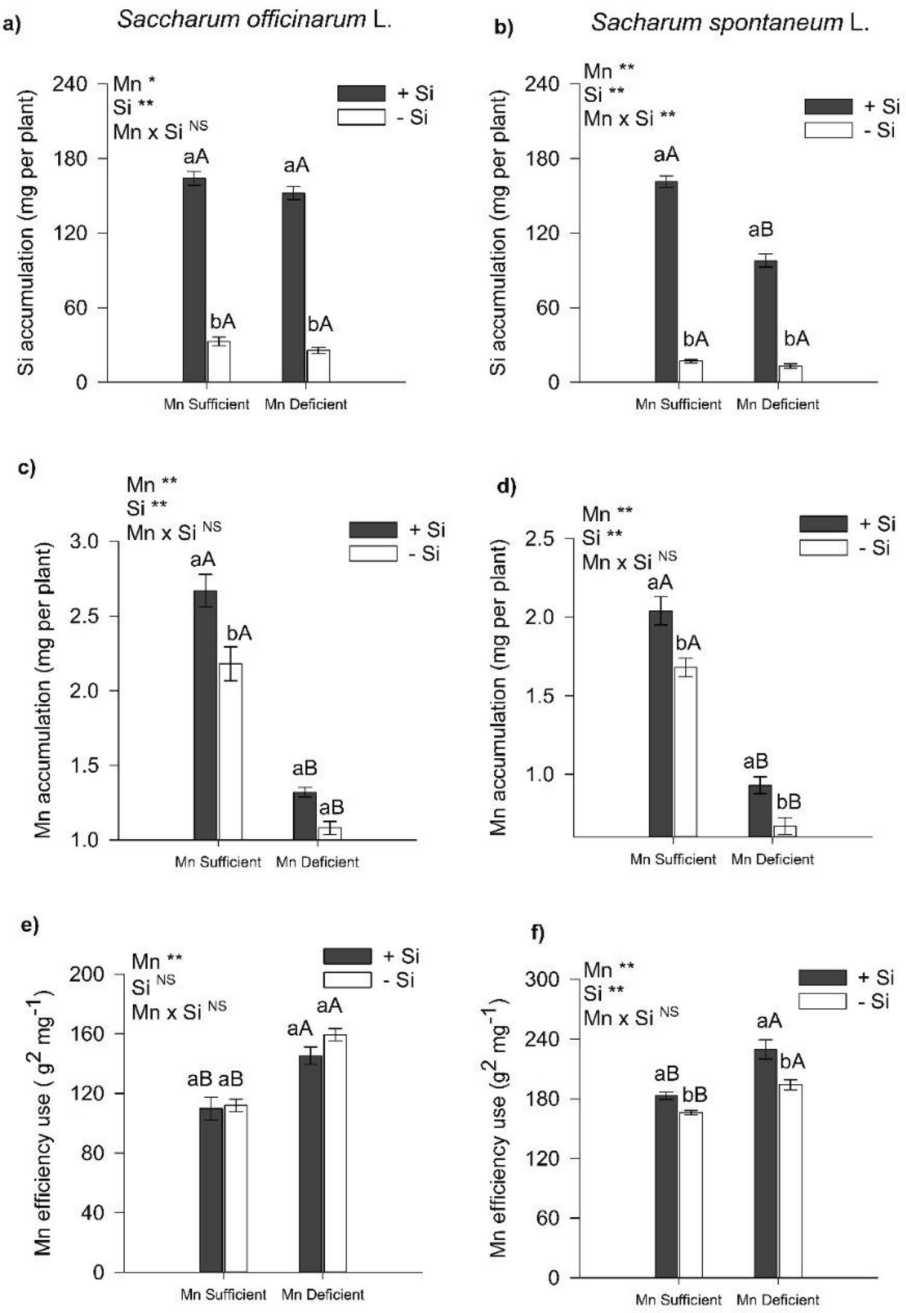
Si (**a**,**b**) and Mn accumulation (**c**,**d**) and Mn use efficiency (**e**,**f**) of sugarcane (*Saccharum officinarum* L.) and energy cane (*Saccharum spontaneum* L.) plants, respectively, grown in Mn-sufficient and deficient nutrient solution in the absence (− Si) and presence (+ Si) of Si. F-test was applied: * (p ≤ 0.05); **(p ≤ 0.01), and *ns* not significant. Different lowercase letters compare Si conditions under the same Mn condition (p < 0.05 by Tukey test), while different uppercase letters compare Mn conditions under the same Si condition (p < 0.05 by Tukey test). Bars represent the mean standard error, n = 5.

The Si supplied via nutrient solution increased its accumulation in sugarcane plants (Fig. 1a) and energy cane plants (Fig. 1b). The change in the plant’s Mn status (deficient or sufficient) does not affect the plant's ability to accumulate Si; however, this only occurred for sugarcane (Fig. 1a). For energy cane in a condition of Mn deficiency, it results in a low accumulation of Si compared to plants with sufficiency of Mn (Fig. 1b).

There was a decrease in Mn accumulation in sugarcane and energy cane under Mn deficiency in relation to Mn sufficiency in plants with the presence of Si and absence of Si (Fig. 1c,d). The presence of Si in the nutrient solution, in relation to the absence of Si, increased the accumulation of Mn by 23% in sugarcane cultivated under Mn sufficiency. There was no change in plants under Mn deficiency (Fig. 1c). For energy cane, the presence of Si, in relation to its absence in the nutrient solution, increased by 40% and 21% the accumulation of Mn in plants grown in nutrient solution deficient in Mn and in nutrient solution sufficient in Mn, respectively (Fig. 1d).

The presence of Si in the nutrient solution, in relation to the absence of Si, did not affect Mn use efficiency in sugarcane cultivated under Mn deficiency and sufficiency (Fig. 1e). However, for energy cane, the presence of Si in the nutrient solution, in relation to the absence of Si, increased the plant's Mn use efficiency by 10% and 18% when grown in nutrient solution with Mn sufficiency and deficiency, respectively (Fig. 1f).

### Lipid peroxidation (MDA), SOD and GPOX activity, and phenol content

**Figure 2:**
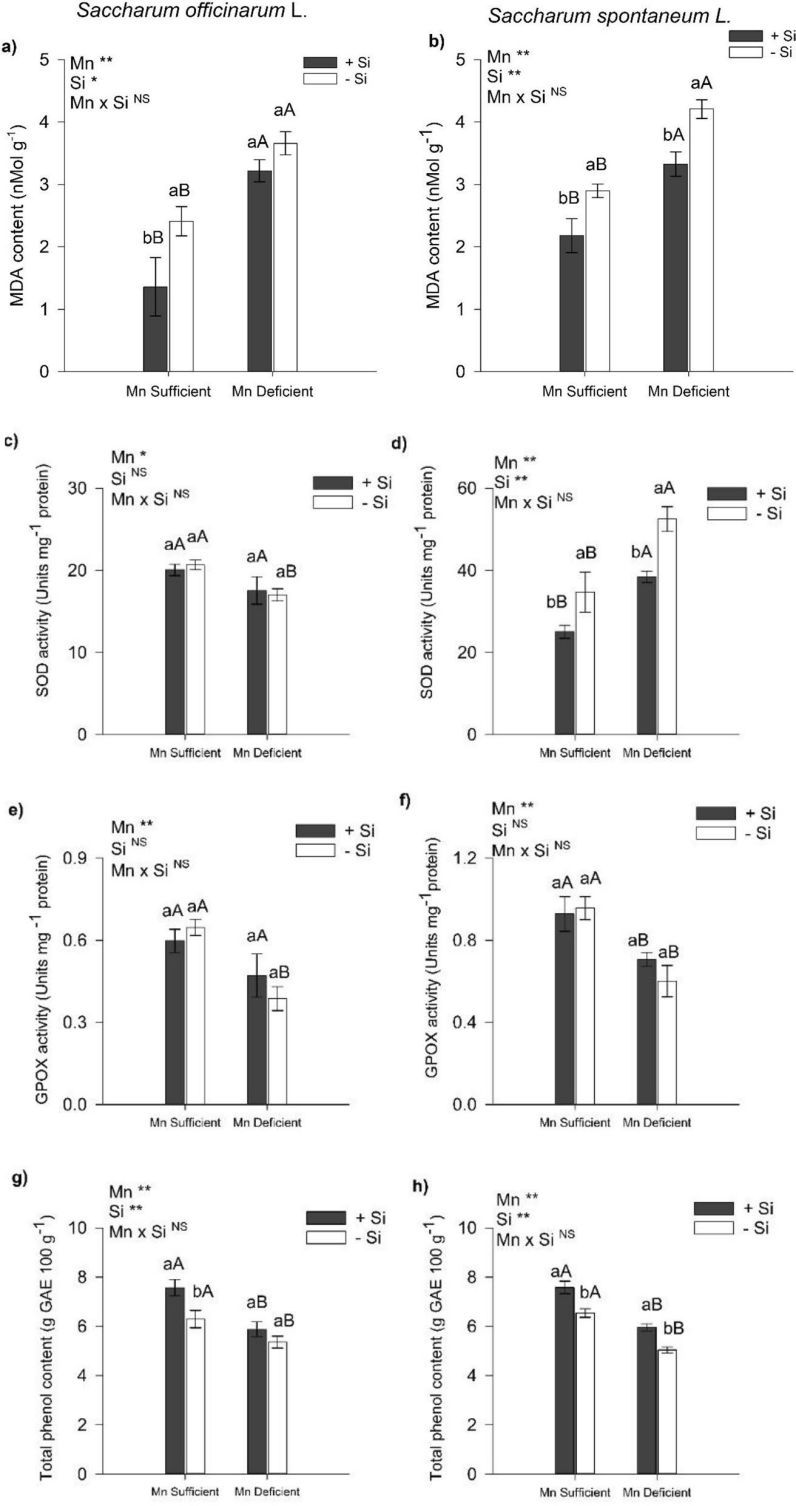
MDA content (**a**,**b**), superoxide dismutase (SOD) activity (**c**,**d**), guaiacol peroxidase (GPOX) activity (**e**,**f**), and total phenol content (**g**, **h**) of leaves of sugarcane (*Saccharum officinarum* L.) and energy cane (*Saccharum spontaneum* L.) plants, respectively, grown in Mn-sufficient and deficient nutrient solution in the absence (− Si) and presence (+ Si) of Si. F-test was applied: * (*p* ≤ 0.05); **(*p* ≤ 0.01), and *ns* not significant. Different lowercase letters compare Si conditions under the same Mn condition (*p* < 0.05 by Tukey test), while different uppercase letters compare Mn conditions under the same Si condition (*p* < 0.05 by Tukey test). Bars represent the mean standard error, n = 5.

There was an increase in MDA content in Mn-deficient sugarcane and energy cane compared to plants grown in a nutrient solution sufficient in Mn. The presence of Si in the nutrient solution in relation to its absence decreased the MDA content (by 44%) in sugarcane plants with Mn sufficiency in the nutrient solution (Fig. 2a). In energy cane plants cultivated in the presence of Si, in relation to the absence of Si, there was a decrease in MDA content by 25% and 21% in plants sufficient in Mn and in plants deficient in Mn, respectively (Fig. 2b).

Sugarcane plants cultivated in nutrient solution under Mn deficiency in relation to plants sufficient in Mn and in the absence of Si in the nutrient solution presented lower SOD and GPOX activities (Fig. 2c,e). Energy cane plants deficient in Mn, in relation to Mn sufficient plants cultivated in the absence of Si in the nutrient solution, showed a higher SOD activity. However, the presence of Si in the nutrient solution, in relation to the absence of Si in energy cane, reduced the SOD activity in plants under Mn sufficiency (28%) and under Mn deficiency (27%) (Fig. 2d). There was a decrease in GPOX activity in energy cane plants cultivated in nutrient solution under Mn deficiency in relation to Mn-sufficient plants that did not receive Si. The presence of Si in the nutrient solution in relation to the absence of Si in it did not affect GPOX activity in energy cane in both Mn conditions (deficient and sufficient) (Fig. 2f).

There was a decrease in the content of total phenols in sugarcane and energy cane plants cultivated under Mn deficiency in relation to plants sufficient in Mn and those that did not receive Si. There was an increase in the content of total phenols by 20% in sugarcane plants cultivated in nutrient solution with the presence of Si in the nutrient solution in relation to the absence of Si in plants sufficient in Mn (Fig. 2g). In energy cane plants, the presence of Si in the nutrient solution, in relation to the absence of Si, increased the contents of total phenols in Mn-deficient plants (18%) and in Mn-sufficient plants (16%) (Fig. 2h).

### Pigments content and PSII quantum efficiency

**Figure 3:**
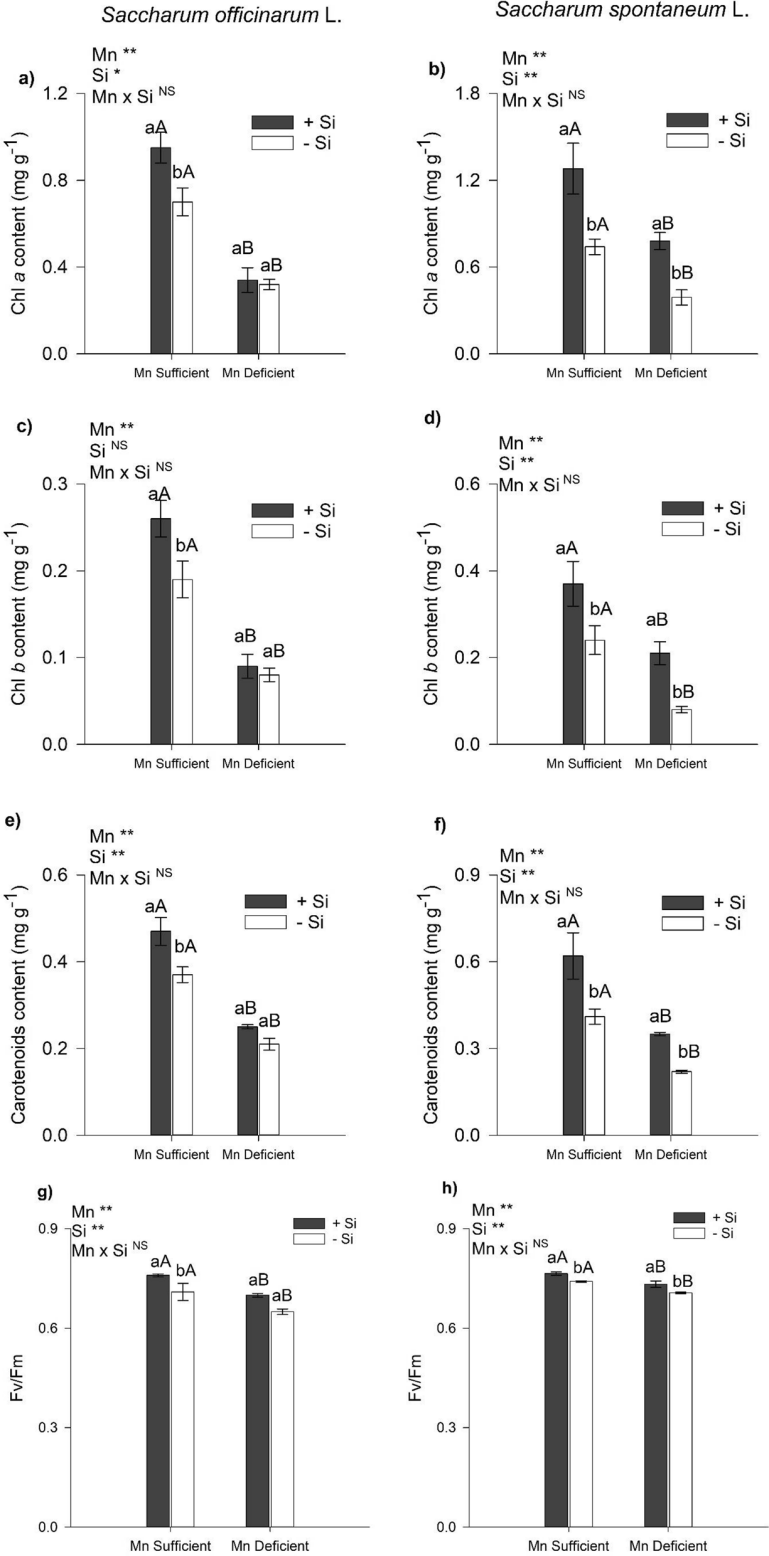
Content of chlorophyll *a* (**a**,**b**), chlorophyll *b* (**c**,**d**), carotenoids (**e**,**f**), and photosystem II quantum efficiency (**g**,**h**) of sugarcane (*Saccharum officinarum* L.) and energy cane (*Saccharum spontaneum* L.) plants, respectively, grown in Mn-sufficient and deficient nutrient solution in the absence (− Si) and presence (+ Si) of Si. F-test was applied: *(*p* ≤ 0.05); **(*p* ≤ 0.01), and *ns* not significant. Different lowercase letters compare Si conditions under the same Mn condition (*p* < 0.05 the Tukey test), while different uppercase letters compare Mn conditions under the same Si condition (*p* < 0.05 by Tukey test). Bars represent the mean standard error, n = 5.

There was a decrease in pigment content (Chl *a, b,* carotenoids) in sugarcane and energy cane plants (Fig. 3a–f) cultivated in nutrient solution under Mn deficiency in relation to sufficient Mn and those that did not receive Si in the nutrient solution. The presence of Si in the nutrient solution, in relation to its absence, increased the contents of Chl *a*, Chl *b*, and carotenoids (27%) in sugarcane plants under Mn sufficiency (Fig. 3a,c,e). In energy cane, the presence of Si in the nutrient solution, in relation to the absence of Si, increased the contents of Chl *a* (Fig. 3b), Chl *b* (Fig. 3d), and carotenoids by 64% and 51% (Fig. 3f) in Mn-deficient and Mn-sufficient plants, respectively.

There was a decrease in PSII quantum efficiency in sugarcane and energy cane plants cultivated in nutrient solution deficient in Mn in relation to a Mn-sufficient solution and those that did not receive Si in the nutrient solution (Fig. 3g,h). The presence of Si in the nutrient solution, in relation to the absence of Si, increased the PSII quantum efficiency in sugarcane plants under Mn sufficiency (Fig. 3g). In energy cane, the presence of Si in the nutrient solution, in relation to the absence of Si, increased the PSII quantum efficiency in Mn deficiency and in Mn sufficiency (Fig. 3h).

### Protein content and growth

**Figure 4:**
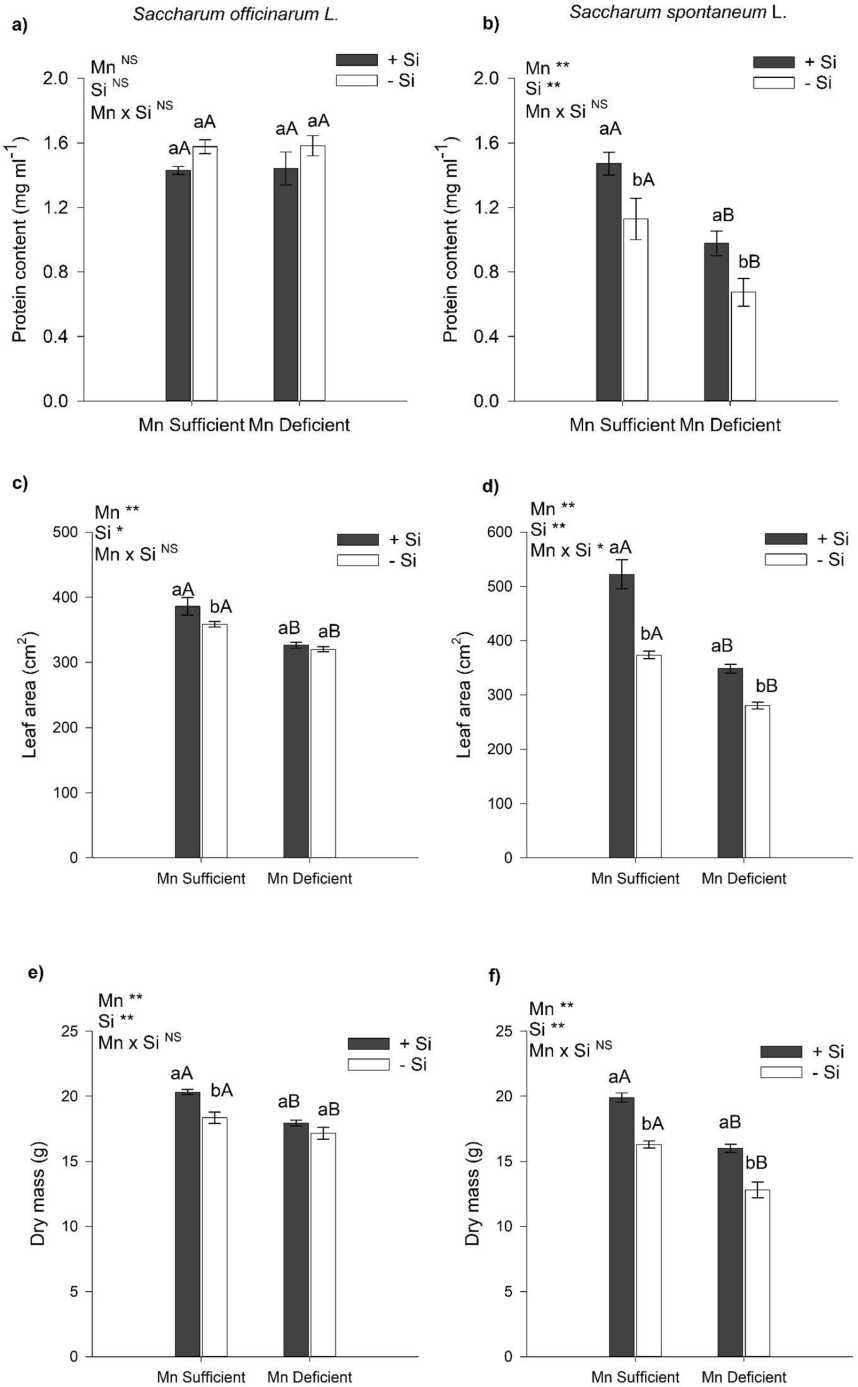
Protein content (**a**,**b**), leaf area (**c**,**d**), and total dry mass (**e**,**f**) of sugarcane (*Saccharum officinarum* L.) and energy cane (*Saccharum spontaneum* L.) plants, respectively, grown in Mn-sufficient and deficient nutrient solution in the absence (− Si) and presence (+ Si) of Si. F-test was applied: * (*p* ≤ 0.05); **(*p* ≤ 0.01), and *ns* not significant. Different lowercase letters compare Si conditions under the same Mn condition (*p* < 0.05 by Tukey test), while different uppercase letters compare Mn conditions under the same Si condition (*p* < 0.05 by Tukey test). Bars represent the mean standard error, n = 5.

The sufficiency or deficiency conditions of Mn and the absence and presence of Si in the nutrient solution did not affect protein contents in sugarcane (Fig. 4a). There was a decrease in protein content in energy cane plants cultivated under Mn deficiency in relation to Mn-sufficient plants that did not receive Si in the nutrient solution. The presence of Si in the nutrient solution, in relation to the absence of Si, increased the protein content in energy cane under Mn deficiency and in Mn sufficiency (Fig. 4b).

There was a decrease in leaf area and dry mass of sugarcane plants and energy cane plants deficient in Mn in relation to plants sufficient in Mn and those that did not receive Si (Fig. 4c,d). The presence of Si, in relation to its absence in the nutrient solution, increased the sugarcane leaf area in plants grown in a sufficient nutrient solution of Mn (Fig. 4c). In energy cane, the presence of Si in the nutrient solution, in relation to the absence of Si, increased the leaf area in plants deficient in Mn and in plants sufficient in Mn (Fig. 4d).

There was an increase in the dry mass of sugarcane plants sufficient in Mn (11%) when cultivated in nutrient solution with Si in relation to the absence of Si (Fig. 4e). In energy cane, the presence of Si in relation to the absence of Si in the nutrient solution provided increases of 22 and 25% in plant dry mass in Mn sufficiency and Mn deficiency, respectively (Fig. 4f).

## Discussion

Plants of both species grown in Mn-deficient nutrient solution with or without Si reduced Mn absorption, consequently causing biological damage to plants.

Manganese deficiency with no addition of Si to the nutrient solution promotes an increase in ROS, as observed in sorghum^5^, resulting in an increase in MDA content in both studied species. This plant response may be associated with cellular damage caused by the excess of ROS production in the metabolism and/or by a lower capacity of ROS elimination by the defense system, leading to formation of MDA, which is a secondary metabolite resulting from the degradation of hydroperoxides of polyunsaturated fatty acids^36^ and an indicator of oxidative stress. This has not yet been reported for sugarcane, but it was observed in Mn-deficient sorghum^5^ and corn plants^4^.

Our results also show that enzymatic responses under Mn deficiency may be different depending on species despite a clear occurrence of oxidative stress due to increased lipid peroxidation. SOD activity in Mn-deficient sugarcane plants was low in the absence of Si, a fact also reported in other studies^5,10^. Mn is a cofactor of the Mn-SOD enzyme in plants and a key enzyme in the O_2_^•-^ dismutation process in H_2_O_2_^11,12^. The lack of this micronutrient may decrease this enzyme activity in the metabolism and consequently O_2_^•-^ accumulation in the cells, thus causing lipid degradation.

However, SOD activity in energy cane increased in Mn-deficient plants. Mn is one of the SOD cofactors, and therefore the total SOD activity may vary depending on species, reflecting the regulation of other isoforms of this enzyme, such as Cu/Zn-SOD and Fe-SOD, increasing in turn the total enzyme activity^8,37^. In addition, an increase in SOD activity in Mn-deficient plants suggests a higher ROS production^38^. Therefore, it is important to consider that the total SOD activity can be different depending on species even under the same type of stress.

Similar as SOD, peroxidases are part of the defense system components. They control the H_2_O_2_ content produced in the metabolism by SOD catalysis and in a non-enzymatic manner^39^. Mn-deficient sugarcane and energy cane plants presented a decrease in the peroxidase activity studied (GPOX). The low activity of another peroxidase (APX) under Mn deficiency has already been reported and related to one of the main responses to the deprivation of this nutrient^10,38^. This low enzyme activity may have contributed to the lipid peroxidation observed in Mn-deficient energy cane plants due to excessive H_2_O_2_ accumulation, which is a substrate of this enzyme and causes cell degradation^39^ because SOD activity was high in this species.

In this scenario, Mn deficiency reduced the content of photosynthetic pigments in both studied species due to oxidative stress related to a direct ROS action, which causes thylakoid disorganization^13^ and cell membrane degradation^40^. In addition, Mn deficiency decreased PSII quantum efficiency, as it causes a reduction in the complexes of this nutrient at the PSII core, inducing destabilization and disintegration of this photosystem^14^ and increasing ^1^O_2_ formation^9^, which can in turn increase the degradation of chlorophylls. Also, reductions in the content of carotenoids and phenols increase the damage to chlorophylls, as they may cause accumulations of ^1^O_2_, an active molecular oxygen, and H_2_O_2_, thus potentiating oxidative damage^39^.

On the other hand, Mn deficiency did not affect protein contents in sugarcane plants. However, energy cane plants showed an important decrease in protein contents. Mn activates RNA polymerase and the glycolytic enzymes of proteins^15,16^, with reflexes on protein synthesis. However, according to^12^, protein synthesis is not necessarily impaired and may even increase in Mn-deficient tissues, as^41^ observed. The decrease in protein contents of Mn-deficient sugarcane plants may be the result of oxidative stress due to protein oxidation by ROS, suggesting a marked nutritional damage related to the lack of Mn in this species.

The damage Mn deficiency caused to plants, mainly in structures indispensable for photosynthesis, such as pigments and the PSII, with reflections on the oxidative metabolism, justifies the growth losses of both species given the decrease in leaf area and dry mass production. The effects that Mn deficiency causes on photosynthesis are mainly responsible for the impacts it exerts on plant growth^5,14,42^.

The physiological and biochemical damage of Mn deficiency, although not yet reported for sugarcane and energy cane, indicates the importance of this micronutrient for these species, thus confirming a need for strategies such as the use of Si to mitigate such effects. Our study presents unprecedented answers to these issues by evaluating possible mechanisms of action of Si and its relations with Mn.

Initially, the Si supply in the nutrient solution was efficient to increase Si accumulation in the sugarcane and energy cane grown in a nutrient solution deficient or not in Mn. The high amount of accumulated Si in these plants may be explained by the improved root absorption capacity of this element by this crop^26,43^, which is caused by the presence of specific carriers of this element, which controls its absorption^44^. The ability of *S. spontaneum* L. plants to accumulate Si, not yet reported, confirms that this species has a high Si absorption, similar as that of *S. officinarum* L.

The increase in Si absorption favored Mn absorption in both studied species regardless of deficiency or sufficiency in this micronutrient. This result is important because it contributes to understanding the role of Si in Mn absorption. It is one of the reasons that this element favors Mn nutrition whether in sufficiency or deficiency. The greater the absorption of Mn by the plant, the greater the expectation in increasing the plant's nutritional and physiological responses to this micronutrient. Therefore, this is a new finding for these species. There are reports only for other grasses, such as sorghum under Mn deficiency^18^ and corn and wheat without deficiency of this micronutrient^33^. This beneficial Si effect on Mn absorption could be related to the regulation of the H^+^-ATPase activity^28,45,46^, as well as to specific Mn transporters such as IRT1 and AtNramp^30^, which generate an electrochemical gradient for nutrient absorption, including Mn carriers in the membrane^29,31^. They were identified for sugarcane^32^.

The increase in Mn absorption caused by Si addition to Mn-sufficient sugarcane and Mn-sufficient and Mn-deficient energy cane could explain the increase in total phenol content. According to^6^, it occurs because Mn associates with the biosynthesis of phenolic compounds by activating enzymes involved in the formation of secondary metabolites that are precursors to phenols^23^. Some studies have reported that Si increases the amount of this antioxidant compound^34^. Also, the increased Mn due to Si provided increases of another important antioxidants (carotenoids), as Mn activates the enzyme phytoene synthase, which acts in the biosynthesis of isoprenoids, precursors of this pigment^47^. Therefore, Mn and Si may act together to increase the contents of these two non-enzymatic antioxidants in both studied species.

Thus, the increase in phenols provided by Si aids the defense system of plants subjected to different stresses^48,49^ and plants under no stress^34^. Phenols act in balancing the antioxidant system, with a direct elimination of active molecular oxygen and H_2_O_2_, thus inhibiting lipid peroxidation^39^. Moreover, Mn-deficient and sufficient energy cane plants with Si presented reductions in SOD activity, but with a low lipid peroxidation. This may be related to a reduction in the O_2_^•-^ produced in the presence of the beneficial element in response to the best Fv/Fm ratio of these plants. Therefore, it suggests low losses of electrons in the transport chain in the photosystems II and I in chloroplasts because these electrons reduce molecular oxygen (O_2_) and form O_2_^•-^^50^, along with the action of other antioxidant compounds, and not with a direct action of SOD, in this case.

Other studies have reported reductions in SOD activity in the presence of Si^51–53^, including sugarcane^54^, and that they vary according to species. Despite this, there were reductions in oxidative stress promoted by Si associated with the action of other components linked to enzymatic and non-enzymatic antioxidant metabolism.

Carotenoids play an essential role in protecting the photosynthetic apparatus, contributing to decrease the formation of singlet oxygen (^1^O_2_). Singlet oxygen is formed by a deficiency in the dissipation of energy by the PSII^39^. Therefore, the action of Si, with mechanisms to reduce the production of ROS (O_2_^**·**−^, H_2_O_2,_^1^O_2_) and to increase the antioxidant compounds that reduce the concentrations of H_2_O_2_ (phenols) and ^1^O_2_ (carotenoids), may have contributed to reduce the lipid peroxidation observed. The regulation of antioxidant enzyme activity has been reported only in sorghum plants grown under Mn deficiency, modulating the activity of the enzymes SOD and ascorbate peroxidase (APX)^5^. However, the Si action in the enzyme and non-enzymatic antioxidant system occur in other stresses; recent studies have indicated Si benefits in reducing lipid peroxidation^55,56^.

Silicon did not benefit the enzymatic complexes SOD and GPOX in sugarcane. However, the reduction in lipid peroxidation in Mn-sufficient plants may have been the result of an action of the phenols and carotenoids studied in this species because they affect important ROS that degrades cells, thus improving metabolism and reducing natural damage to plant cells with no Mn deficiency. Also, Si may have aided other defense system complexes that have not been studied, but that may be the focus of further studies.

The effects of Si described above have not yet been reported for *S. officinarum* L. and *S. spontaneum* L. This reveals the benefits of Si in reducing MDA in the leaves of the species evaluated as a result of the modulation of the antioxidant system. These actions may have delayed the natural degradation of chlorophyll, which resulted in an increase in pigments in leaves of sugarcane and energy cane under Mn sufficiency. However, the presence of Si in Mn-deficient sugarcane plants also decreased chlorophyll degradation. This occurs because Si promotes an increase in Mn accumulation in the plant. According to^13^, Mn decreases the damage to chloroplasts because it is involved with lipid synthesis. Also, ROS accumulation may have been suppressed in chloroplasts due to an improvement in the antioxidant system, which may in turn have reduced the oxidation of existing pigments, which explains the increase in this pigment in plants under deficiency stress. Gonzalo et al.^57^ already described a low chlorophyll degradation by Si in soybeans and cucumber and^58^ for cucumber.

The application of silicon in energy cane plants promoted an increase in Mn use efficiency and protein content under Mn sufficiency and deficiency. In plants sufficient in Mn, the physiological benefits provided by this micronutrient, such as increased pigments and PSII efficiency, may have increased photosynthesis, reflecting on the increase in protein synthesis. In Mn-deficient plants, in addition to physiological benefits, the reduction of oxidative stress provided by Si reduced damage to cellular components, including proteins. This justifies increases in protein contents. In rice plants under cadmium stress, Si regulated six categories of proteins, including those involved with protein synthesis^59^, thus showing the benefits of Si for protein synthesis under stress conditions. Therefore, the nutritional, physiological, and biochemical improvements provided high conversions of photoassimilates into dry mass and into protein content^60,61^, as observed for other grasses with and without Mn deficiency stress^5,17^.

The presence of Si in sugarcane did not affect the Mn use efficiency and the protein content of plants. Use efficiency is partly modulated by the capacity and genetic characteristics that are intrinsic to the species or cultivar^62^. Similarly, the action of Si on soluble protein depends on the condition under which the plants grow and species metabolism. In our study, Si did not act on Mn deficiency in sugarcane. There are reports that the benefits of Si in increasing protein contents are observed in plants under higher stress levels^63,64^ and that are also associated with reductions in ROS. Therefore, it occurred because of different species and metabolic variations.

The benefits promoted by the application of Si to the nutrient solution, especially for energy cane under Mn deficiency or not and sugarcane under Mn sufficiency, reflected, in a same proportion, in the growth of these species due to the increase in leaf area and dry mass. Si benefits plants mainly under stress conditions^25^, a fact that occurred in energy cane but not in sugarcane. In this case, the deficiency in this micronutrient was more severe in energy cane due to a more drastic decrease in the dry mass of this species (22%) compared to sugarcane (7%). Therefore, Si was not relevant to mitigate the slight Mn deficiency in sugarcane, as this element, under this condition, did not increase total phenols, carotenoids, and the activity of the antioxidant enzymes SOD and GPOX, which could act to maintain ROS homeostasis and improve the pigment content, the PSII quantum efficiency, and Mn use efficiency, and proteins, thus reflecting on plant growth.

However, there was a more severe Mn deficiency observed in energy cane. The Si increased the accumulation of Mn, which reflected in physiological gains shown by the increase in pigment contents and PSII quantum efficiency, in addition to biochemical gains by reducing lipid peroxidation (MDA) and increases in non-enzymatic antioxidants (phenols and carotenoids). This resulted in a greater use efficiency of Mn, protein contents, and production of dry matter. These are benefits of Si to dry matter contents of Mn-deficient plants, as verified for rice^65^ and sorghum^5^.

The beneficial responses of Si application to a non-stressed plant were evident in this study for both species due to an increase in plant growth explained by increases in Mn accumulation, Mn use efficiency, and pigments. This result reinforces the indication that Si may enhance the response of plants without stress due to an increased Mn accumulation, as verified for corn and wheat^33^, total phenols in wheat^34^, and PSII quantum efficiency and chlorophyll content in rice^66^.

Therefore, in general, Si can mitigate the damage caused by Mn deficiency in energy cane plants and improve the responses of both species to the micronutrient supply by increasing Mn absorption and the production of antioxidant compounds, pigments, and PSII quantum efficiency. This is in line with the hypothesis of this study, which proposed such effects of Si on both species and that it could be more evident in plants under stress, depending on species. Thus, these facts are confirmed in our study, as the responses were different according to species.

Finally, the beneficial relationship between Si and Mn was unveiled, and the indication of this beneficial element was proposed for the cultivation of sugarcane without Mn deficiency and energy cane with or without Mn deficiency to increase the sustainability of the cultivation of these species through a better management of micronutrient supply.

## Conclusion

The sugarcane and energy cane are sensitive to Mn deficiency as it causes important biological damage. At the same time, both species are responsive to the application of this micronutrient by presenting improvements in the antioxidant and physiological metabolism and an increase in total phenols, GPOX activity, pigments, and PSII quantum efficiency.

The attenuation of effects of manganese deficiency by silicon depends on species and stress level, with a greater benefit for *Saccharum spontaneum* L. The action of this beneficial element reflects on increases in the accumulation of Mn, which reduces oxidative stress by increasing antioxidant compounds, regulating the activity of SOD, and increasing the chlorophyll content, PSII quantum efficiency, use efficiency of Mn, and growth. It is thus a tool to improve the performance of this species in soils deficient in Mn.

## Material and methods

### Experimental conditions and plant growth

Two experiments were carried out using the sugarcane *S. officinarum* L., variety RB 966928, and the energy cane *S. spontaneum* L., type I, variety VX3, in a greenhouse at the São Paulo State University (UNESP), Jaboticabal Campus, Brazil, from January to July 2019.

Pre-sprouted seedlings from mini-cuttings (5 cm long) with one bud were planted in a polypropylene tray filled with fine vermiculite. Si was applied via fertigation after the full emergence of shoots at intervals of four days for 50 days and via a complete *Hoagland* and *Arnon*^67^ solution with a change in the Fe-EDTA source to Fe-EDDHA. Subsequently, the plants received the nutrient solution with low Mn content (0.1 µmol L^−1^) for 32 days to induce deficiency. The seedlings were then transplanted to 1.5-dm^3^ polypropylene pots filled with washed sand.

### Experimental design and treatments

The treatments were applied from the seedling transplant. They consisted of a 2 × 2 factorial design with five replications arranged in random blocks for each species. The treatments consisted of plants grown under Mn sufficiency (20.5 µmol L^−1^) and deficiency (0.1 µmol L^−1^) associated with the absence or presence of Si (2.0 mmol L^−1^). The Si source was sodium and potassium silicate stabilized with sorbitol (SiNaKE) at 107.9 g L^−1^ of Si and 16.44 g L^−1^ of K_2_O. The *Hoagland and Arnon*^67^ nutrient solution, with a modification in Mn concentration, was used. The pH value of the nutrient solution was adjusted to 5.5 ± 0.5 using solutions of 1.0 mol L^−1^ HCl or 1.0 mol L^−1^ NaOH. The potassium concentration of the nutrient solution for the treatments without Si was adjusted with potassium chloride. The average relative air humidity and temperature were measured inside the greenhouse for both experiments during the growing period: 15 and 62% of minimum and maximum relative air humidity and 17 and 36 °C of minimum and maximum temperature.

Biochemical and physiological evaluations were carried out on plants at the end of the experiments, 58 days after the treatments were applied.

### Analyses

#### Mn and Si accumulation and Mn use efficiency

The Mn content was determined by digesting 0.1 g of dry and ground material from shoots and roots in 1.5 mL of nitro-perchloric solution 2:1 under heating blocks at an initial temperature of 80 °C, increased each 30 min until reaching 210 °C. After that, the reading was taken of the diluted extract in water in atomic absorption according to the method proposed in^68^.

Si content was determined in shoots and roots using 0.1 g of dry and ground material, which were added to 50-mL polyethylene screw-cap centrifuge tubes. The samples were moistened with 2 mL of hydrogen peroxide (H_2_O_2_) after washing the sides of the tube to remove the sample. The tube was tightly capped and placed in an oven at 95 °C. After 30 min, the tubes were removed, and 4 mL of 50% sodium hydroxide (NaOH) were added to warm samples. The sample tubes were then gently vortexed, capped tightly, and returned to the oven (95 °C) for four hours according to the methodology described in^69^. The Si concentration was determined by colorimetry using 1 mL extract plus 19 mL of water, 1 mL of HCl (1:1), and 2 mL ammonium molybdate. After 5 min, 2 mL oxalic acid were added. The reading performed by a spectrophotometer at 410 nm as described in^70^. The total Mn and Si accumulation was obtained by the sum of the product of the dry mass and the content of elements in shoots and roots. Mn use efficiency in shoots was determined according to^71^ based on Mn accumulation.

#### Lipid peroxidation (MDA)

Lipid peroxidation was determined using 0.3 g of the first fully expanded leaf 58 days after transplanting (DAT) and treatment application, according to the methods described in^72^. The concentration of malondialdehyde (MDA) equivalents was determined by reading on a spectrophotometer between 535 and 600 nm and the data was calculated based on the extinction coefficient of 1.55 × 10^−5^ mol^−1^ cm^−1^, with MDA results expressed in nMol g^−1^ of fresh matter^73^.

#### Protein extraction

The total soluble protein was extracted using 1.0 g of the first fully developed leaf collected at 58 DAT and homogenized in a cooled mortar and pestle containing 100 mM potassium phosphate buffer (pH 7.5), 1 mM ethylenediaminetetraacetic acid (EDTA), 3 mM DL-dithiothreitol, and 5% (w/v) insoluble polyvinylpolypyrrolidone in a 3:1 volume/fresh mass ratio^74^. The material was centrifuged at 10,000×*g* for 30 min, and the supernatant was stored at − 80 °C for later determination of activities of the SOD and GPOX enzymes. Protein concentration was determined using the^75^ method with bovine serum albumin as a standard, expressed as mg mL^−1^ protein.

#### Superoxide dismutase (SOD) activity

The SOD activity (SOD, EC 1.15.1.1) was determined in a spectrophotometer, as described in^76^, and the reaction was conducted in a reaction chamber (box) under the lighting of a 15 W fluorescent lamp at 25 °C. The reading was performed at 560 nm, and the SOD activity was expressed as U mg^−1^ protein.

#### Guaiacol peroxidase (GPOX) activity

The GPOX activity (GPOX, EC 1.11.1.7) was determined in a mixture of phosphate citrate buffer at pH 5.0 (0.2 M dibasic sodium phosphate:0.1 M citric acid), 0.5% guaiacol, and extract. The activity was evaluated by monitoring the absorbance at a wavelength of 450 nm^77^.

#### Total phenol content

Total phenolic compounds were extracted using 0.1 g of fresh fully expanded leaves collected at 59 DAT and concentrated methanol in a water bath at 25 °C. After extraction, a colorimetric reaction of total phenols was induced with the 2 N Folin-Ciocalteu reagent, allowing reacting for three minutes, and 20% sodium carbonate, allowing reacting for two hours. In the end, the absorbance was read on a spectrophotometer at a wavelength of 765 nm, while the content was determined using a standard curve with gallic acid, expressed as g gallic acid equivalent (GAE) 100 g^−1^^78^.

#### Chlorophyll *a*, Chlorophyll *b*, and carotenoids

The pigment content was determined using 0.027 g of leaf discs taken from the middle third of the leaf blade completely expanded at 59 DAT, according to the methodology proposed by^79^. Readings at 663 nm for chlorophyll *a* (Chl *a*), 647 nm for chlorophyll *b* (Chl *b*), and 470 nm for carotenoids were performed on a Beckman DU 640 spectrophotometer. The contents were defined based on fresh mass.

#### PSII quantum efficiency

The PSII quantum efficiency (Fv/Fm) was determined by measuring chlorophyll fluorescence using a fluorimeter (Opti-sciences—Os30P). For this, the sampled region was submitted to the dark for adaptation at least 30 min before the excitation of the red light pulse of 1 s. Measurements were performed between 7:30 and 8:30 a.m. on the middle third of leaf + 1 (first complete leaf with visible sheath) at 58 DAT.

#### Growth

The leaf area of sugarcane and energy cane was measured at 62 DAT using a leaf area measuring device (L-3100, Li-Cor, EUA). The value was given in cm^2^. The dry mass was determined by cutting the plants at the substrate level and separating shoots from roots to compose the total dry mass. The fractions were washed with detergent solution (0.1% v/v), acid solution with 1.0 mol L^−1^ HCl (0.3% v/v), and deionized water. Subsequently, the samples were stored in paper bags and dried in a forced air circulation oven at 65 ± 2 °C until constant mass. The dry mass of the whole plant was obtained in grams.

### Statistical analysis

The data were checked for normality (Shapiro—Wilk test) and homogeneity of variances (Levene’s test) in software libre R 4.0.3 and subjected to analysis of variance by F-test using the, the means were compared by Tukey test at 5% probability, and the SAS statistical program (Cary, NC, USA). The interactions were sliced even when not significant and the elaborated graphs in SigmaPlot 14.0 (Systat Software, Inc, San Jose, CA).

### Ethical approval

The authors confirm that the handling of the plants is accordance with the Declaration of IUCN Policy on Research Involving Endangered Species and the Convention on Trade in Endangered Species of Wild Fauna and Flora.

## References

[1] Husted S (2009). Manganese deficiency leads to genotype-specific changes in fluorescence induction kinetics and state transitions. Plant Physiol..

[2] Hue N, Mai Y (2002). Manganese toxicity in watermelon as affected by lime and compost amended to a Hawaiian acid Oxisol. HortScience.

[3] Mousavi SR, Shahsavari M, Rezaei M (2011). A general overview on manganese (Mn) importance for crops production. Aust. J. Basic Appl. Sci..

[4] Zhao HQ (2014). Oxidative stress of maize roots caused by a combination of both salt stress and manganese deprivation. Cereal Res. Comm..

[5] de Oliveira RLL, de Mello Prado M, Felisberto G, Checchio MV, Gratão PL (2019). Silicon mitigates manganese deficiency stress by regulating the physiology and activity of antioxidant enzymes in sorghum plants. J. Soil Sci. Plant Nutr..

[6] Lidon FC, Barreiro M, Ramalho J (2004). Manganese accumulation in rice: Implications for photosynthetic functioning. J. Plant Physiol..

[7] Rodrigues WS, Pereira YC, de Souza ALM, Batista BL, Lobato AK (2020). Alleviation of oxidative stress induced by 24-epibrassinolide in soybean plants exposed to different manganese supplies: UpRegulation of antioxidant enzymes and maintenance of photosynthetic pigments. J. Plant Growth Regul..

[8] Shenker M, Plessner OE, Tel-Or E (2004). Manganese nutrition effects on tomato growth, chlorophyll concentration, and superoxide dismutase activity. J. Plant Physiol..

[9] Krieger-Liszkay A, Fufezan C, Trebst A (2008). Singlet oxygen production in photosystem II and related protection mechanism. Photosynth. Res..

[10] Yu Q, Osborne L, Rengel Z (1998). Micronutrient deficiency changes activities of superoxide dismutase and ascorbate peroxidase in tobacco plants. J. Plant Nutr..

[11] Millaleo M, Reyes-Diaz M, Ivanov AG, Mora ML, Alberdi M (2010). Manganese as essential and toxic element for plants transport, accumulation and resistance mechanisms. J. Soil Sci. Plant Nutr..

[12] Broadley M, Brown P, Cakmak I, Rengel Z, Zhao F, Marschner P (2012). Function of nutrients: Micronutrients. Mineral Nutrition of Higher Plants.

[13] Qu C (2012). Effects of manganese deficiency and added cerium on photochemical efficiency of maize chloroplasts. Biol. Trace Elem. Res..

[14] Schmidt SB, Jensen PE, Husted S (2016). Manganese deficiency in plants: The impact on photosystem II. Trends Plant Sci..

[15] Ness PJ, Woolhouse HW (1980). RNA synthesis in Phaseolus chloroplasts: I. Ribonucleic acid synthesis in chloroplast preparations from *Phaseolus vulgaris* l. Leaves and solubilization of the RNA polymerase. J. Exp. Bot..

[16] Strasser R (2007). A unique β1,3-galactosyltransferase is indispensable for the biosynthesis of N-glycans containing Lewis a structures in *Arabidopsis thaliana*. Plant Cell.

[17] Moradtalab N (2018). Silicon improves chilling tolerance during early growth of maize by effects on micronutrient homeostasis and hormonal balances. Front. Plant Sci..

[18] Oliveira KS, de Mello Prado R, Guedes VH (2020). Leaf spraying of manganese with silicon addition is agronomically viable for corn and sorghum plants. J. Soil Sci. Plant Nutr..

[19] Orlando Filho J, Câmara GMS, Oliveira EAM (1993). Calagem e adubação da cana-de-açúcar. Produção de Cana-de-açúcar.

[20] Mellis EV, Quaggio JA, Becari GRG, Teixeira LAJ, Cantarella H, Dias FLF (2016). Effect of micronutrients soil supplementation on sugarcane in different production environments: Cane plant cycle. Agron. J..

[21] Carvalho-Netto OV (2014). The potential of the energy cane as the main biomass crop for the cellulosic industry. Chem. Biol. Technol. Agric..

[22] Matsuoka S, Rubio LCS, Khan M, Khan I (2019). Energy cane: A sound alternative of a bioenergy crop for tropics and subtropics. Sugarcane Biofuels.

[23] Burnell JN, Graham RD, Hannam RJ, Uren NC (1988). The biochemistry of manganese in plants. Manganese in Soils and Plants.

[24] Malavolta E (2006). Manual de Nutrição de Plantas.

[25] Epstein E (2009). Silicon: Its manifold roles in plants. Ann. Appl. Biol..

[26] Savant NK, Korndörfer GH, Datnoff LE, Snyder GH (1999). Silicon nutrition and sugarcane production: A review. J. Plant Nutr..

[27] Mitani N, Ma JF (2005). Uptake system of silicon in different plant species. J. Exp. Bot..

[28] Xu CX, Ma YP, Liu YL (2015). Effects of silicon (Si) on growth, quality and ionic homeostasis of aloe under salt stress. S. Afr. J. Bot..

[29] Zhang J (2017). The role of the plasma membrane H+-ATPase in plant responses to aluminum toxicity. Front. Plant Sci..

[30] Bao Q (2021). Silicon combined with foliar melatonin for reducing the absorption and translocation of Cd and As by *Oryza sativa* L. in two contaminated soils. J. Environ. Manag..

[31] Socha AL, Guerinot ML (2014). Mn-euvering manganese: The role of transporter gene family members in manganese uptake and mobilization in plants. Front. Plant Sci..

[32] Figueira A, Kido EA, Almeida RS (2001). Identifying sugarcane expressed sequences associated with nutrient transporters and peptide metal chelators. Genet. Mol. Biol..

[33] Greger M, Landberg T, Vaculík M (2018). Silicon influences soil availability and accumulation of mineral nutrients in various plant species. Plants..

[34] Mendonça AO, Tavares LC, Brunes AP, Monzón DLR, Villela FA (2013). Acúmulo de silício e compostos fenólicos na parte aérea de plantas de trigo após a adubação silicatada. Biosci. J..

[35] Hörtensteiner S, Kräutler B (2011). Chlorophyll breakdown in higher plants. Biochim. Biophys. Acta..

[36] Geng A (2018). Silicon improves growth and alleviates oxidative stress in rice seedlings (*Oryza sativa* L.) by strengthening antioxidant defense and enhancing protein metabolism under arsanilic acid exposure. Ecotoxicol. Environ. Saf..

[37] Yu Q, Rengel Z (1999). Micronutrient deficiency influences plant growth and activities of superoxide dismutases in narrow-leafed lupins. Ann. Bot..

[38] Tewari RK, Kumar P, Sharma PN (2013). Oxidative stress and antioxidant responses of mulberry (*Morus alba*) plants subjected to deficiency and excess of manganese. Acta Physiol. Plant.

[39] Sharma P, Jha AB, Dubey RS, Pessarakli M (2012). Reactive oxygen species, oxidative damage, and antioxidative defense mechanism in plants under stressful conditions. J. Bot..

[40] Papadakis IE (2007). Mn-induced changes in leaf structure and chloroplast ultrastructure of *Citrus volkameriana* (L.) plants. J. Plant Physiol..

[41] Lerer M, Bar-Akiva A (1976). Nitrogen constituents in manganese-deficient lemon leaves. Physiol. Plant..

[42] Gong X (2011). Cerium relieves the inhibition of photosynthesis of maize caused by manganese deficiency. Biol. Trace Elem. Res..

[43] Yan G, Nikolic M, Ye M, Xiao Z, Liang Y (2018). Silicon acquisition and accumulation in plant and its significance for agriculture. J. Integr. Agric..

[44] Mitani N, Yamaji N, Ma JF (2008). Identification of maize silicon influx transporters. Plant Cell Physiol..

[45] Liang Y, Zhang W, Chen Q, Liu Y, Ding R (2006). Effect of exogenous silicon (Si) on H+-ATPase activity, phospholipids and fluidity of plasma membrane in leaves of salt-stressed barley (*Hordeum vulgare* L.). Environ. Exp. Bot..

[46] Kim Y (2014). Silicon mitigates heavy metal stress by regulating P-type heavy metal ATPases, *Oryza sativa* low silicon genes, and endogenous phytohormones. BMC Plant Biol..

[47] Wilkinson RE, Ohki K (1988). Influence of manganese deficiency and toxicity on isoprenoid syntheses. Plant Physiol..

[48] Gomes FB, Moraes JC, Santos CD, Goussain MM (2005). Resistance induction in wheat plants by silicon and aphids. Sci. Agric..

[49] Vega I (2019). Silicon improves the production of high antioxidant or structural phenolic compounds in barley cultivars under aluminum stress. Agronomy.

[50] Pospíšil P (2012). Molecular mechanisms of production and scavenging of reactive oxygen species by photosystem II. Biochim. Biophys. Acta.

[51] Šimková L, Fialová I, Vaculíková M, Luxová M (2016). The effect of silicon on the activity and isozymes pattern of antioxidative enzymes of young maize roots under zinc stress. SILICON.

[52] Kim Y-H, Khan AL, Waqas M, Lee I-J (2017). Silicon regulates antioxidant activities of crop plants under abiotic-induced oxidative stress: A review. Front. Plant Sci..

[53] Delavar K, Ghanati F, Behmanesh M, Zare-Maivan H (2018). Physiological parameters of silicon-treated maize under salt stress conditions. SILICON.

[54] Bezerra BKL, Lima GPP, dos Reis AR, Silva MA, Camargo MS (2019). Physiological and biochemical impacts of silicon against water deficit in sugarcane. Acta Physiol. Plant..

[55] Ur-Rahman S (2020). Zain, M. Silicon and its application methods improve physiological traits and antioxidants in *Triticum aestivum* (L.) under cadmium stress. J. Soil Sci. Plant Nutr..

[56] Ur-Rahman S (2021). Alleviatory effects of Silicon on the morphology, physiology, and antioxidative mechanisms of wheat (*Triticum aestivum* L.) roots under cadmium stress in acidic nutrient solutions. Sci. Rep..

[57] Gonzalo MJ, Lucena JJ, Hernández-Apaolaza L (2013). Effect of silicon addition on soybean (*Glycine max*) and cucumber (*Cucumis sativus*) plants grown under iron deficiency. Plant Physiol. Biochem..

[58] Bityutskii N, Pavlovic J, Yakkonen K, Maksimović V, Nikolic M (2014). Contrasting effect of silicon on iron, zinc and manganese status and accumulation of metal-mobilizing compounds in micronutrient-deficient cucumber. Plant Physiol. Biochem..

[59] Nwugo CC, Huerta AJ (2011). The effect of silicon on the leaf proteome of rice (*Oryza sativa* L.) plants under cadmium-stress. J. Proteome Res..

[60] Zhu Z, Wei G, Li J, Qian Q, Yu J (2004). Silicon alleviates salt stress and increases antioxidant enzymes activity in leaves of salt-stressed cucumber (*Cucumis sativus* L.). Plant Sci..

[61] Fleck AT (2010). Silicon enhances suberization and lignification in roots of rice *(Oryza sativa)*. J. Exp. Bot..

[62] Baligar VC, Fageria NK, He ZL (2001). Nutrient use efficiency in plants. Commun. Soil Sci. Plant Anal..

[63] Gong HJ, Chen KM, Zhao ZG, Zhou WJ (2008). Effects of silicon on defense of wheat against oxidative stress under drought at different developmental stages. Biol Plant..

[64] Latef AAA, Tran LSP (2016). Impacts of priming with silicon on the growth and tolerance of maize plants to alkaline stress. Front. Plant Sci..

[65] Timotiwu B, Nurmauli N, Yulianti P (2017). Application of manganese and silica through leaves and their effect on growth and yield of rice field in village of Sinar Agung, Sub-district of PulauPanggung, District of Tanggamus, Lampung Province, Indonesia. J. Agric. Sci..

[66] Van Bockhaven J (2015). Primary metabolism plays a central role in moulding silicon-inducible brown spot resistance in rice. Mol. Plant Pathol..

[67] Hoagland DR, Arnon DI (1950). The Water-Culture Method for Growing Plants Without Soil.

[68] Bataglia OC, Furlani AMC, Teixeira JPF, Furlani PR, Gallo JR (1983). Métodos de análise química de plantas. Boletim Técnico (78).

[69] Kraska JE, Breitenbeck GA (2010). Simple, robust method for quantifying silicon in plant tissue. Commun. Soil Sci. Plant Anal..

[70] Korndörfer GH, Pereira HS, Nolla A (2004). Análise de Silício no Solo, Planta e Fertilizante.

[71] Siddiqi MY, Glass AD (1981). Utilization index: A modified approach to the estimation and comparison of nutrient utilization efficiency in plants. J. Plant Nutr..

[72] Heath RL, Packer L (1968). Photoperoxidation in isolated chloroplasts: I. Kinetics and stoichiometry of fatty acid peroxidation. Arch. Biochem. Biophys..

[73] Gratão PL (2012). Biochemical dissection of diageotropica and never ripe tomato mutants to Cd-stressful conditions. Plant. Physiol. Biochem..

[74] Boaretto LF (2014). Water stress reveals differential antioxidant responses of tolerant and non-tolerant sugarcane genotypes. Plant Physiol. Biochem..

[75] Bradford MM (1976). A rapid and sensitive method for the quantitation of microgram quantities of protein utilizing the principle of protein-dye binding. Anal. Biochem..

[76] Cembrowska-Lech D, Koprowski M, Kępczyński J (2015). Germination induction of dormant *Avena fatua* caryopses by KAR1 and GA3 involving the control of reactive oxygen species (H_2_O_2_ and O_2_·−) and enzymatic antioxidants (superoxide dismutase and catalase) both in the embryo and the aleurone layers. J. Plant Physiol..

[77] Gomes-Junior RA (2006). Nickel elicits a fast antioxidante response in *Coffea arabica* cells. Plant Physiol. Biochem..

[78] Singleton VL, Rossi JA (1965). Colorimetry of total phenolics with hosphomolybdic-phosphotungstic acid reagents. Am. J. Enol. Vitic..

[79] Lichtenthaler HK (1987). Chlorophylls and carotenoids: Pigments of photosynthetic biomembranes. Methods Enzymol..

